# By activating matrix metalloproteinase-7, shear stress promotes chondrosarcoma cell motility, invasion and lung colonization

**DOI:** 10.18632/oncotarget.3274

**Published:** 2015-03-14

**Authors:** Pei-Pei Guan, Xin Yu, Jian-Jun Guo, Yue Wang, Tao Wang, Jia-Yi Li, Konstantinos Konstantopoulos, Zhan-You Wang, Pu Wang

**Affiliations:** ^1^ College of Life and Health Sciences, Northeastern University, Shenyang 110819, P. R. China; ^2^ Neural Plasticity and Repair Unit, Wallenberg Neuroscience Center, Department of Experimental Medical Sciences, Lund University, Lund 22184, Sweden; ^3^ Department of Chemical and Biomolecular Engineering, The Johns Hopkins University, Baltimore, Maryland 21218, United States of America; ^4^ Department of Biomedical Engineering, The Johns Hopkins University, Baltimore, Maryland 21218, United States of America; ^5^ Johns Hopkins Institute for NanoBioTechnology, The Johns Hopkins University, Baltimore, Maryland 21218, United States of America; ^6^ Johns Hopkins Physical Sciences-Oncology Center, Center of Cancer Nanotechonology Excellence, The Johns Hopkins University, Baltimore, Maryland 21218, United States of America

**Keywords:** fluid shear stress, cyclic AMP, interleukin-1beta, matrix metalloproteinase 7, chondrosarcoma metastasis

## Abstract

Interstitial fluid flow and associated shear stress are relevant mechanical signals in cartilage and bone (patho)physiology. However, their effects on chondrosarcoma cell motility, invasion and metastasis have yet to be delineated. Using human SW1353, HS.819.T and CH2879 chondrosarcoma cell lines as model systems, we found that fluid shear stress induces the accumulation of cyclic AMP (cAMP) and interleukin-1β (IL-1β), which in turn markedly enhance chondrosarcoma cell motility and invasion via the induction of matrix metalloproteinase-7 (MMP-7). Specifically, shear-induced cAMP and IL-1β activate PI3-K, ERK1/2 and p38 signaling pathways, which lead to the synthesis of MMP-7 via transactivating NF-κB and c-Jun in human chondrosarcoma cells. Importantly, MMP-7 upregulation in response to shear stress exposure has the ability to promote lung colonization of chondrosarcomas *in vivo*. These findings offer a better understanding of the mechanisms underlying MMP-7 activation in shear-stimulated chondrosarcoma cells, and provide insights on designing new therapeutic strategies to interfere with chondrosarcoma invasion and metastasis.

## INTRODUCTION

Matrix metalloproteinase-7 (MMP-7) has a broad proteolytic activity against a number of extracellular matrix (ECM) substrates, such as casein, collagens, fibronectin, laminin, elastin, entactin, cartilage proteoglycans, etc [1]. Accumulating evidence suggests that MMP-7 is necessary for physiological processes associated with tissue remodeling, such as embryonic development, wound healing, trophoblast implantation, and organ morphogenesis [2, 3]. In addition, the enzymatic activity of MMP-7 in degrading ECM potentially contributes to the invasion and metastasis of tumor cells by increasing proliferation, by hydrolyzing the ECM and promoting migration and angiogenesis, and by affecting cell apoptosis [4–6]. Along these lines, Sugita *et al*. [7] reported that the imunnostaining score of MMP-7 increases with the histological grade of chondrosarcomas. MMP-7 may thus serve as a useful indicator in the diagnosis of chondrosarcoma [8]. However, the mechanism of MMP-7 regulation and its biological functions in chondrosarcoma metastasis *in vivo* remain unclear.

The major signaling pathway found to regulate MMP-7 expression in larynx carcinoma is epithelial growth factor receptor (EGFR) signaling pathway [9]. PI3-K/AKT signaling pathway mediated the upregulation of MMP-7 via FoxO1-activating manner in EGF-stimulated Hep-2 cells [9]. Moreover, Shi *et al*. [10] reported that MMP-7 expression was upregulated by activating AP-1 and stat3 in catecholamine-stimulated human gastric cancer cells, which promote the invasion and metastasis of gastric cancer. Apart from AP1 and stat3, the forkhead box transcription factor (FOXC1) was identified to mediate MMP-7 upregulation in human breast cancer cells [11].

Interstitial fluid flow is a relevant mechanical signal in cartilage and bone (patho)physiology [12]. Interstitial fluid flow and associated fluid shear stress induce MMP-1 and MMP-12 expression in human chondrosarcoma cells [13, 14]. Although MMP-7 is elevated in advanced stages of chondrosarcoma disease [7] as well as in shear-activated chondrosarcoma cells [13], the signaling mechanism by which MMP-7 activation may contribute to chondrosarcoma invasion and metastasis has yet to be investigated. We herein demonstrate that fluid shear stress stimulates the synthesis of cAMP and IL-1β, which in turn activate PI3-K/AKT, ERK1/2 and p38 mitogen-activated protein kinase (MAPK)-dependent pathways. PI3-K, ERK1/2 and p38 transactivate the phosphorylation of c-Jun and NF-κB, which in turn bind to the MMP-7 promoter and mediate MMP-7 mRNA and protein synthesis in human shear-stimulated chondrosarcoma cells. We further demonstrate the contribution of MMP-7 activation to lung colonization *in vivo* which may help us gain insights into therapeutic strategies aiming to combat chondrosarcoma metastasis.

## RESULTS

### MMP-7 is upregulated in human chondrosarcoma tissues and shear-activated chondrosarcoma cells

**Figure 1:**
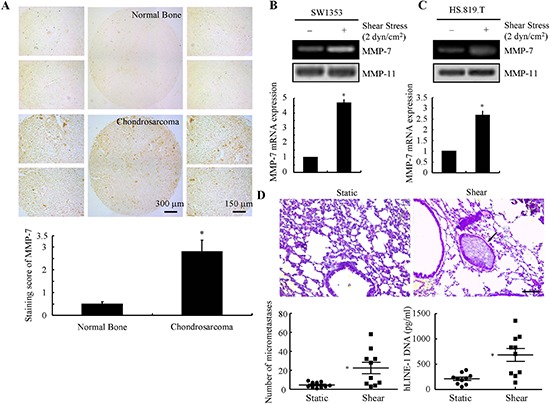
Fluid shear stress activates MMP-7 in human chondrosarcoma cells and promotes their lung colonization *in vivo* **(A)** MMP-7 expression in human chondrosarcoma tissue specimens was determined by immunohistochemical staining using an anti-MMP-7 antibody. Strong immunostaining was detected in ~90% of cancerous tissues, whereas no immunostaining was present in normal bone tissues. SW1353 **(B)** or HS.819.T cells **(C)** were subjected to fluid shear stress (2 dyn/cm^2^) or static conditions (0 dyn/cm^2^) for 48 h. MMP-7 mRNA and protein levels were determined by qRT-PCR and zymography, respectively. GAPDH and MMP-11 total protein amount served for internal control in qRT-PCR and zymography assays, respectively. **(D)** CH2879 cells were exposed to either static or shear (2 dyn/cm^2^) conditions for 48 h. Cells suspended in their corresponding conditioned medium were injected into mice via tail vein (*t* = 0 weeks); the conditioned medium was injected via the tail vein every 3 days for 5 weeks. The right lung lobes from each animal were fixed, stained with hematoxylin and eosin, and examined for signs of lung micrometastases (indicated by arrowheads) (upper panel). Quantification of the number of micrometastases present in lungs of mice following tail vein injection in the absence or presence of static- or shear-conditioned medium as described above; *n* = 10 mice per group (lower left panel). Presence of human DNA quantified in lungs of mice injected with CH2879 chondrosarcoma cells via qPCR of hLINE-1 DNA. *n* = 10 mice per group. Data represent the mean ± S.E. of 3 independent experiments. *p < 0.05 with respect to normal bone tissues or static controls.

Prior work revealed that MMP-7 is detected in human chondrosarcoma but not normal cartilage [7]. Because of the limited number of tissue specimens (a total of 28) examined in the previous study [7], we first wished to confirm these data. Consistent with prior findings [7], MMP-7 immunostaining was markedly elevated (~3-fold) in human chondrosarcoma tissues relative to normal bone controls (Fig. 1A). Given that interstitial fluid flow and associated fluid shear stress are relevant mechanical signals in cartilage and bone (patho)physiology, we next evaluated the effects of fluid shear on MMP-7 expression in human chondrosarcoma cells, using SW1353, HS.819.T and CH2879 chondrosarcoma cell lines as model systems. Our data reveal that cell exposure to a fluid shear stress level of 2 dyn/cm^2^ for 48 h markedly induced the MMP-7 mRNA expression and activity in SW1353 and HS.819.T cells (Figs. 1B, 1C). In light of these observations, we examined whether shear stress has ability to promote lung colonization of human chondrosarcoma cells *in vivo*. To this end, shear-activated CH2879 chondrosarcoma cells along with their shear-conditioned medium were injected via the tail vein into NOD/SCID/IL2 receptor gamma knockout mice; mice were sacrificed 35 days following tail-vein injection. H.E. staining was used to determine the number of micrometastases, whereas qPCR was used to quantify the content of human DNA (and thus tumor colonization) in the lungs of mice. The results reveal that solid tumors were formed in the lungs of mice after 35 days (Fig. 1D). The extent of lung colonization was significantly higher in shear-activated versus static control chondrosarcoma cells. These data suggest that fluid shear stress may potentially contribute to chondrosarcoma invasion and metastasis via induction of MMP-7.

### cAMP and IL-1β regulate the shear-dependent upregulation of MMP-7 via activation of PI3-K/AKT, ERK1/2 and p38 pathways in human chondrosarcoma cells

**Figure 2:**
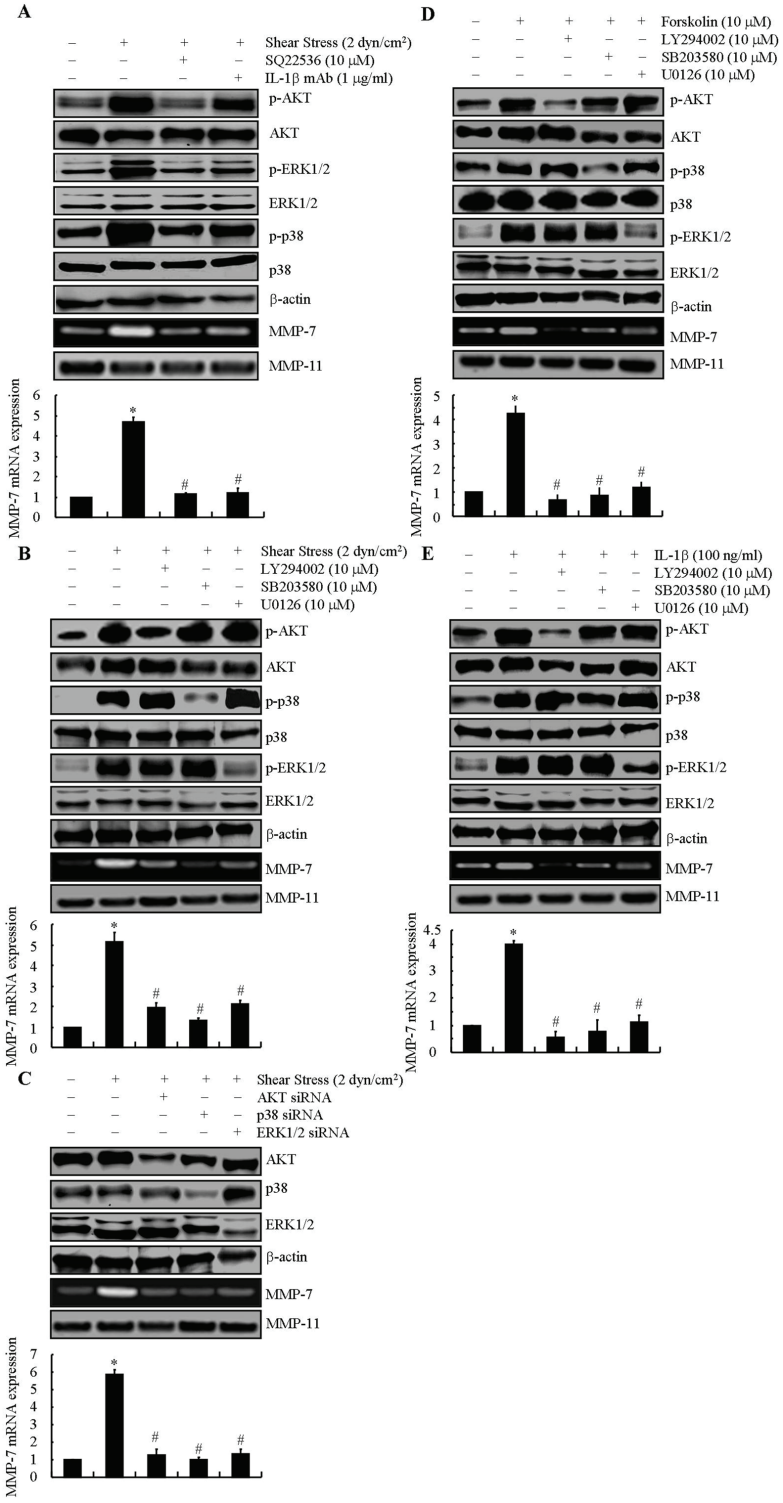
Involvement of cAMP and IL-1β in mediating fluid shear stress to regulate the synthesis of MMP-7 via AKT, ERK1/2 and p38 signaling pathways in SW1353 cells SW1353 cells were subjected to fluid shear stress (2 dyn/cm^2^) or static conditions (0 dyn/cm^2^) in the absence or presence of adenylyl cyclase inhibitor, SQ22536 (10 μM) or IL-1β mAb (1 μg/ml) for 48 h **(A)** In select experiments, SW1353 cells were exposed in shear stress (2 dyn/cm^2^) **(B)**, forskolin (10 μM) **(D)** or IL-1β (100 ng/ml) **(E)** in the absence or presence of LY294002 (10 μM), SB203580 (10 μM) or U0126 (10 μM) for 48 h. In separate experiments, SW1353 cells were transfected with siRNAs targeted to AKT, p38 or ERK1/2 before subjecting to fluid shear stress (2 dyn/cm^2^) **(C)** Phosphorylated AKT, ERK1/2 and p38 are shown by immunoblotting using specific Abs. Equal loading in each lane is ensured by the similar intensities of total AKT, ERK1/2, p38 and β-actin. These western blots are representative of three independent experiments, all revealing similar results. MMP-7 mRNA and protein levels were determined by qRT-PCR and zymography, respectively. GAPDH and MMP-11 total protein amount served for internal control in qRT-PCR and zymography assays, respectively. Data represent the mean ± S.E. of 3 independent experiments. *p < 0.05 *vs*. static- or vehicle-treated control, #*p* < 0.05 vs. shear, forskolin- or IL-1β-treated alone.

We next aimed to delineate the signaling cascade of MMP-7 induction in shear-activated chondrosarcoma cells. Prior work has shown that exogenously added IL-1β upregulates MMP-7 expression in human LNCaP prostate cells and articular chondrocytes [15, 16], whereas cAMP can induce various MMPs in diverse cell types [17]. Interestingly, we recently reported that fluid shear increases the accumulation of both cAMP and IL-1β in human chondrosarcoma cells [14]. Thus, we examined the potential roles of cAMP and IL-1β in MMP-7 expression and activity in sheared SW1353 cells. Incubation of SW1353 chondrosarcoma cells with either an adenylate cyclase inhibitor, SQ22536 (10 μM) or an anti-IL-1β antibody (1 μg/ml) just prior to the onset of shear stress exposure abolished shear-induced MMP-7 mRNA synthesis and activity (Fig. 2A).

Because fluid shear induces MMP-9 synthesis in human chondrocytes via PI3-K- and MAPK-dependent pathways [18], we evaluated the potential role of these signaling pathways in MMP-7 upregulation in shear-activated chondrosarcoma cells. Application of a shear stress level of 2 dyn/cm^2^ for 48 h increased the phosphorylation levels of AKT, ERK1/2 and p38 without altering their respective total levels (Figs. 2A, S1A). Interestingly, the adenylate cyclase inhibitor SQ22536 or an anti-IL-1β antibody alone were effective in suppressing the shear-induced upregulation of AKT, ERK1/2 and p38 phosphorylation, which result in MMP-7 suppression in shear-activated SW1353 chondrosarcoma cells (Fig. 2A). To establish the involvement of PI3-K/AKT, ERK1/2 and p38 signaling pathways in shear-induced MMP-7 activation, experiments were carried out by incubating SW1353 cells with selective PI3-K inhibitor LY294002 (10 μM), or the ERK1/2 inhibitor U0126 (10 μM) or the p38 inhibitor SB203580 (10 μM) just prior to the onset of shear exposure. LY294002, U0126 and SB203580 nearly abolished the phosphorylation of AKT, ERK1/2 and p38, respectively, without affecting their total levels (Figs. 2B, S1B), and markedly diminished shear-induced MMP-7 mRNA expression and activity (Fig. 2B). To validate the involvement of these signaling pathways in MMP-7 regulation in sheared chondrosarcoma cells, experiments were performed by transfecting SW1353 cells with siRNAs targeting AKT, ERK1/2 or p38. These molecular interventions effectively knocked down the protein expression of AKT, ERK1/2 or p38 relative to cells transfected with a scramble siRNA control (Figs. 2C, S1C). Knockdown of AKT, ERK1/2 or p38 markedly repressed MMP-7 mRNA expression and enzymatic activity (Fig. 2C).

To further establish the involvement of cAMP and IL-1β in the signaling cascade regulating MMP-7 activation in sheared SW1353 chondrosarcoma cells, static experiments were carried out by treating cells with forskolin (10 μM), which activates cAMP formation or exogenously added IL-1β (100 ng/ml). Forskolin and IL-1β markedly enhanced the expression and enzymatic activity of MMP-7 (Figs. 2D, 2E) as well as the phosphorylation of AKT, ERK1/2 and p38 (Figs. 2D, 2E, S1D, E). In addition, incubation of SW1353 cells with LY294002 (10 μM), U0126 (10 μM) or SB203580 (10 μM) abolished both forskolin- and IL-1β -dependent MMP-7 upregulation (Figs. 2D, 2E), which is attributed to the ability of these inhibitors to selectively suppress the phosphorylation of AKT, ERK1/2 or p38 in human chondrosarcoma cells (Figs. 2D, 2E). Collectively, our data suggest that fluid shear induces the accumulation of both cAMP and IL-1β, which in turn activate the AKT, ERK1/2 and p38 signaling pathways that are responsible for the elevated MMP-7 expression and enzymatic activity in himan chondrosarcoma cells.

### c-Jun and NF-κB regulate shear-induced MMP-7 mRNA synthesis in human chondrosarcoma cells

**Figure 3:**
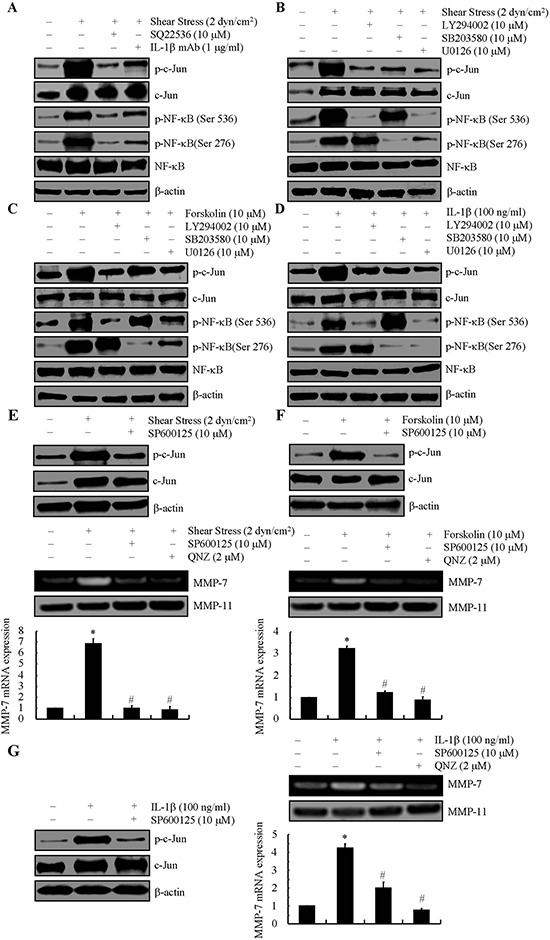
Fluid shear stress activates transcriptional factors, c-Jun and NF-κB, via PI3-K, p38 and ERK1/2 pathways, which result in MMP-7 induction in human SW1353 chondrosarcoma cells SW1353 cells were subjected to fluid shear stress (2 dyn/cm^2^) or static conditions (0 dyn/cm^2^) for 48 h in the absence or presence of either adenylyl cyclase inhibitor, SQ22536 (10 μM), IL-1β mAb (1 μg/ml) **(A)**, PI3-K inhibitor, LY294002 (10 μM), p38 inhibitor, SB203580 (10 μM), ERK1/2 inhibitor, U0126 (10 μM) **(B)** JNK inhibitor, SP600125 (10 μM) or NF-κB inhibitor, QNZ (2 μM) for 48 h **(E)**. In select experiments, SW1353 cells were treated with 10 μM of forskolin **(C, F)** or 100 ng/ml of IL-1β **(D, G)** in the absence or presence of PI3-K inhibitor, LY294002 (10 μM), p38 inhibitor, SB203580 (10 μM), ERK1/2 inhibitor, U0126 (10 μM) (B, D) JNK inhibitor, SP600125 (10 μM) or NF-κB inhibitor, QNZ (2 μM) for 48 h (F, G). The levels of phosphor-c-Jun, phosphor-NF-κB and total levels of c-Jun, NF-κB from cell lysates were determined by western blots using specific Abs. Equal loading in each lane is ensured by the similar intensities of total β-actin. The gels are representative of at least three independent experiments, all revealing similar results (A-G) MMP-7 mRNA and protein levels were determined by qRT-PCR and zymography, respectively. GAPDH and MMP-11 total protein amount served for internal control in qRT-PCR and Zymography assays, respectively (E-G) Data represent the mean ± S.E. of 3 independent experiments. *p < 0.05 *vs*. static- or vehicle-treated control, #*p* < 0.05 *vs.* shear, forskolin- or IL-1β-treated alone.

We next sought to decipher the critical transcription factor(s) responsible for MMP-7 synthesis in sheared chondrosarcoma cells. The promoter of *mmp-7* gene contains several consensus sequences, including those for AP-1 and NF-κB [19, 20]. Because of the elevated AKT, ERK1/2 and p38 phosphorylation levels in shear stress-, forskolin- and IL-1β-stimulated chondrosarcoma cells, we examined the potential contributions of AKT, ERK1/2 and p38 to regulating the activities of c-Jun and NF-κB. Application of fluid shear to human SW1353 cells induces phosphorylation of c-Jun at Ser 63 and p65 at both Ser 536 and Ser 276 (Figs. 3A, S2A). Cell treatment with SQ22536 (10 μM) or an anti-IL-1β antibody (1 μg/ml) repressed the shear-induced phosphorylation of c-Jun and NF-κB down to basal levels (Figs. 3A, S2A). Akin inhibitory effects on the phosphorylation of c-Jun were noted in shear stress-, forskolin- or IL-1β- activated SW1353 cells that were pre-treated with LY294002 (10 μM), SB203580 (10 μM) or U0126 (10 μM) (Figs. 3B–3D, S2B-D). Remarkably, the PI3-K inhibitor LY294002 (10 μM) nearly abrogated p65 phosphorylation at Ser 536, while leaving intact the phosphorylation at Ser 276 in shear-, forskolin or IL-1β- activated SW1353 cells (Figs. 3B–3D, S2B-D), whereas the p38 inhibitor SB203580 (10 μM) had the reverse effect on p65 phosphorylation (Figs. 3B–3D, S2B-D). It is also noteworthy that the ERK1/2 inhibitor U0126 (10 μM) suppressed the phosphorylation of p65 at both sites (Figs. 3B–3D, S2B-D). These data suggest the potential involvement of c-Jun and NF-κB in regulating the synthesis of MMP-7 in shear-activated chondrosarcoma cells. To validate this hypothesis, cells were first incubated with the JNK inhibitor SP600125 (10 μM). This treatment nearly abrogated the induction of MMP-7 in shear-, forskolin- and IL-1β-activated SW1353 cells, presumably by markedly attenuating the phosphorylation of c-Jun at Ser 63 (Figs. 3E–3G, S2E). Incubation of SW1353 cells with the NF-κB inhibitor quinazoline (QNZ) (2 μM) also abolished the expression and enzymatic activity of MMP-7 in shear stress-, forskolin- and IL-1β-stimulated SW1353 cells (Figs. 3E–3G, S2E).

**Figure 4:**
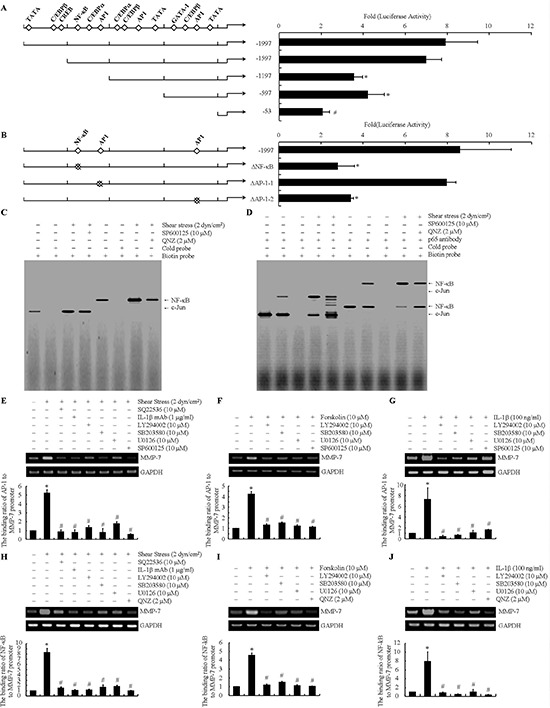
NF-κB and c-Jun are identified as the essential transcriptional factors for MMP-7 synthesis in human chondrosarcoma cells **(A, B)** SW1353 cells were transfected with *mmp-7* promoter expression plasmids or mutation constructs before being subjected to fluid shear stress (2 dyn/cm^2^) for 48 h. Luciferase activities, normalized to renilla luciferase activities, were measured by using the Dual-Luciferase Reporter Assay Kit. Data represent the mean ± S.E. of three independent experiments. *p < 0.05 with respect to pMMP-7(−1997/+39). In select experiments, nuclear extracts were isolated, and NF-κB- and c-Jun-specific DNA-protein complex formation was determined by EMSA **(C, D).** In separate experiments, cross-linked chromatin was immunoprecipitated using an anti-c-Jun **(E-G)** or anti-p65 antibody **(H-J)** In the ChIP assays, the anti-RNA polymerase II antibody was used as a positive control. DNA purified from immunoprecipitated (*IP*) and preimmune (*Input*) specimens was subjected to qPCR amplification using primers for the *mmp-7* promoter. All experiments are representative of three independent experiments, all revealing similar results (E-J) The data represent the means ± S.E. of three independent experiments. *p < 0.05 with respect to the static or vehicle treatment control. #*p* < 0.05 compared with fluid shear stress, forskolin or IL-1β treatment alone.

To establish the involvement of c-Jun and NF-κB in the regulation of shear-induced MMP-7 mRNA synthesis, a series of MMP-7 promoter constructs were generated using the luciferase reporter plasmid, pGL3-basic vector (Fig. 4A, left panel). As a first step, SW1353 cells were transiently transfected with a construct encompassing the 5′-flanking region of the human MMP-7 gene from −1997 to +39 bp prior to their exposure to fluid shear for 48 h. Shear stress induced a pronounced (~8 fold) increase in the MMP-7 promoter activity in SW1353 cells (Fig. 4A). A similar 8-fold upregulation was detected upon transfection of cells with plasmids containing deletions from −1997 to −1597 bp (−1597/+39), thereby suggesting that DNA region between −1997 and −1597 bp upstream of the transcriptional start site is not crucial to the induction of shear-induced MMP-7 promoter activity (Fig. 4A). However, subsequent deletion from −1597 to −1197 bp (−1597/+60) markedly diminished the luciferase activity (Fig. 4A). Bioinformatics analysis of the consensus sequence in (−1597/−1197) region revealed the presence of a NF-κB site, which may be responsible for shear-induced MMP-7 synthesis. Indeed, introduction of a point mutation into one of the aforementioned NF-κB binding site (−1507/−1498) significantly diminished shear-activated luciferase activity relative to the reported wild type (Fig. 4B). Using similar experimental methods, we identified another AP-1 binding site (−68/−58), which is important for shear-induced MMP-7 transcription (Figs. 4A, 4B).

The potential involvement of NF-κB and c-Jun in the induction of MMP-7 in shear-activated chondrocytes was next disclosed by gel and supershift assay. Incubation of nuclear extracts from sheared *versus* untreated SW1353 chondrosaroma cells with the biotinylated c-Jun or NF-κB probe leads to the formation of the c-Jun or NF-κB specific DNA-protein complex (Fig. 4C). Furthermore, incubating nuclear extracts from shear-activated SW1353 cells with an anti-c-Jun or anti-p65 antibody prior to the addition of c-Jun or NF-κB probe results in a marked supershift of the complex (Fig. 4D). Both the formation of the c-Jun or NF-κB-specific DNA-protein complex and the supershift are inhibited by treating T/C-28a2 chondrocytes with the selective JNK inhibitor, SP600125 (10 μM) or NF-κB inhibitor QNZ (2 μM) (Figs. 4C, 4D). Finally, ChIP assays were performed to confirm the binding of phosphorylated NF-κB or c-Jun to its putative site on the *mmp-7* promoter. Application of fluid shear (2 dyn/cm^2^) to SW1353 cells for 48 h induced a 5–8 fold increase in the binding of c-Jun and NF-κB to the AP-1 and NF-κB binding sites, respectively, of the *mmp-7* promoter (Figs. 4E, 4H). Treatment of SW1353 cells with SQ22536 (10 μM) or an anti-IL-1β antibody (1 μg/ml) suppressed the shear-induced binding of c-Jun and NF-κB to *mmp-7* promoter down to base levels (Figs. 4E, 4H). Furthermore, incubation of SW1353 cells with the PI3-K inhibitor LY294002 (10 μM), or the ERK1/2 inhibitor U0126 (10 μM) or the p38 inhibitor SB203580 (10 μM) were effective in markedly repressing the binding of c-Jun and NF-κB to the *mmp-7* promoter (Figs. 4E, 4H). More importantly, the JNK inhibitor SP600125 (10 μM) or NF-κB inhibitor QNZ (2 μM) abolished the shear-induced binding of c-Jun and NF-κB to the AP-1 and NF-kB binding sites, respectively, of the *mmp-7* promoter (Figs. 4E, 4H). Similar observations were made using forskolin- or IL-1β-stimulated human chondrosarcoma cells (Figs. 4F, 4G, 4I, 4J). Taken together, these data provide evidence for the pivotal roles of c-Jun and NF-κB transcriptional factors in the regulation of shear-induced MMP-7 expression.

### Shear-induced MMP-7 promotes chondrosarcoma cell motility and invasion

**Figure 5:**
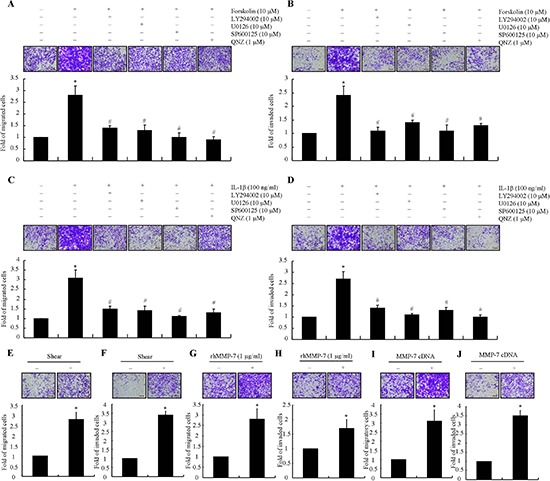
MMP-7 enhances the migration and invasion of human chondrosarcoma cells SW1353 cells were treated with forskolin (10 μM) **(A, B)**, or IL-1β (100 ng/ml) **(C, D)** in the absence or presence of PI3-K inhibitor, LY294002 (10 μM), p38 inhibitor, SB203580 (10 μM), ERK1/2 inhibitor, U0126 (10 μM), JNK inhibitor, SP600125 (10 μM) or NF-κB inhibitor, QNZ (2 μM). In select experiments, SW1353 cells were exposed to either static or shear (2 dyn/cm^2^) for 48 h. Cells suspended in their corresponding conditioned medium were then subjected to transwell experimens **(E, F)**. In separate experiments, SW1353 cells were incubated with rhMMP-7 (1 μg/ml) or vehicle control before being seeded to the transwell device **(G, H)**. In distinct experiments, SW1353 cells were transfected with either MMP-7 cDNA or the empty vector before being seeded to transwell device **(I, J)**. *p < 0.05 with respect to static-, vehicle-treated or vector-transfected control cells. #*p* < 0.05 as compared with forskolin or IL-1β treated cells.

**Figure 6:**
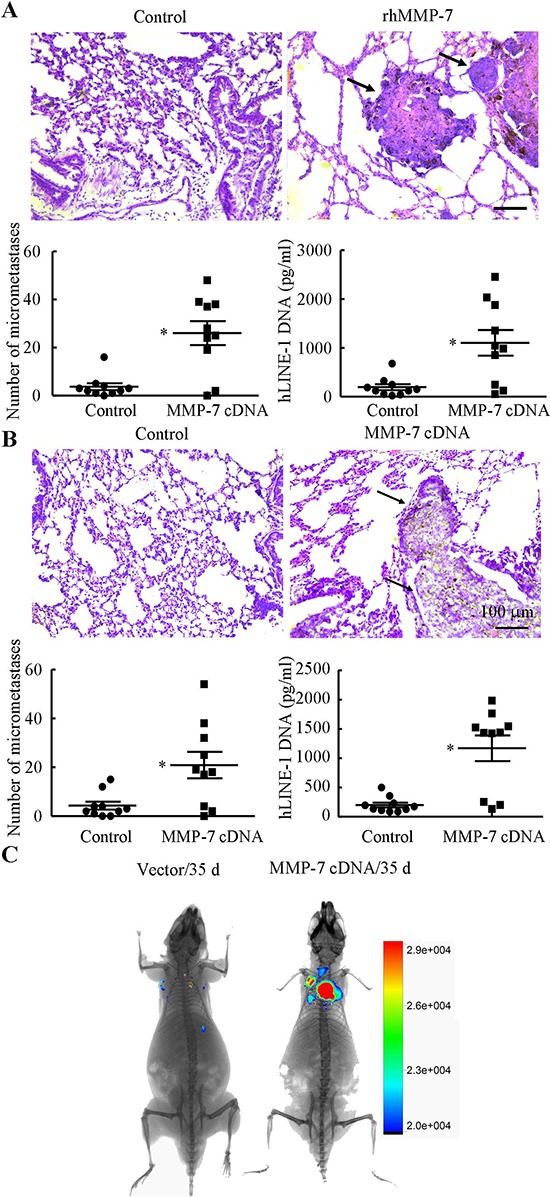
MMP-7 promotes lung colonization *in vivo* **(A)** Mice were injected via the tail vein with either rhMMP-7 (10 μg/ml) or vehicle control at the day of the cell injection (*t* = 0 weeks) and weekly thereafter for 5 weeks. **(B)** In select, experiments, mice were injected with CH2879 cells transfected with either MMP-7 cDNA or the empty vector, *n* = 7–10 mice per group (A, B upper panels) The right lung lobes from each animal were fixed, stained with hematoxylin and eosin, and examined for signs of lung micrometastases and representative histology of lungs following tail vein injection were shown (indicated by arrowheads). (A, B lower panels) The number of micrometastases present in lungs of mice following tail vein injection was quantified in the absence or presence of rhMMP-7 treatment or MMP-7 cDNA plasmid transfection. In addition, content of human DNA was quantified in lungs of mice injected with CH2879 chondrosarcoma cells via qPCR of hLINE-1 DNA. Data represent the mean ± S.E. of 10 independent experiments. *p < 0.05 with respect to static-, vehicle-treated or vector-transfected control cells. In separate experiments, CH2879 cells transfected with MMP-7 cDNA-mcherry plasmid was injected to NGS mice and the fluorescence was scanned using Bruker *in vivo* imaging systems (MS FX PRO, Carestream, U.S.A) **(C)** Images are representative of six independent experiments, all revealing similar results.

**Figure 7:**
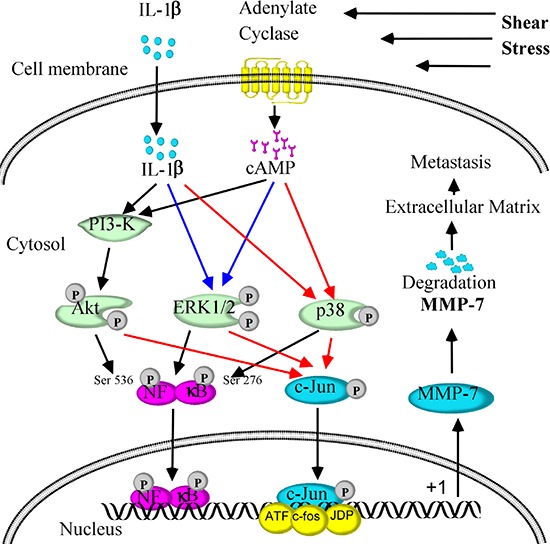
Signaling cascades involved in MMP-7 regulation in response to fluid shear stress, which in turn mediates chondrosarcoma metastasis Fluid shear stress (2 dyn/cm^2^) induces cAMP and IL-1β production and release in human SW1353 chondrosarcoma cells. Elevated levels of cAMP and IL-1β stimulate the activity of PI3-K/AKT, p38 and ERK1/2 pathways, which in turn upregulate MMP-7 synthesis in a c-Jun- and NF-κB-dependent manner. Transactivation of MMP-7 mediates chondrosarcoma migration and invasion, thereby contributing to chondrosarcoma metastasis.

Prior work has suggested that MMP-7 correlates with the degree of malignancy in human chondrosarcoma [7]. We and others have reported the involvement of MMP-7 in the migration and invasion of different tumor cell types [7, 8, 21]. We herein sought to delineate the mechanism by which fluid shear stress potentiates chondrosarcoma cells motility and invasion. In view of our observations showing that endogenous cAMP and IL-1β regulate shear-induced MMP-7 activation, we examined the potential effects of exogenously added forskolin (10 μM) and IL-1β (100 ng/ml) on SW1353 cell motility and invasion. Both forskolin and IL-1β augmented SW1353 cell motility and invasion as shown in Transwell assays (Figs. 5A–5D). Interestingly, incubation of SW1353 cells with the PI3-K inhibitor LY294002 (10 μM), the ERK1/2 inhibitor U0126 (10 μM), the p38 inhibitor SB203580 (10 μM), the JNK inhibitor SP600125 (10 μM) or the NF-kB inhibitor QNZ (2 μM) abrogated the ability of forskolin or IL-1β (100 ng/ml) to augment SW1353 cells migration and invasion (Figs. 5A–5D). To further establish the relationship between shear stress and tumor migration and invasion, shear conditioned medium was collected and used as chemoatractant. The results reveal that sheared medium markedly increased the migration and invasion of human SW1353 cells (Figs. 5E, 5F). In light of these findings, we also assessed the potential contribution of MMP-7 to the migration and invasion of human chondrosarcoma cells. As a first step, we treated human SW1353 cells with rhMMP-7 (1 μg/ml). The results indicate that treatment of SW1353 cells with rhMMP-7 increased their motility and invasion *in vitro* (Figs. 5G, 5H). Of note, MMP-7 overexpression by MMP-7 cDNA plasmid transfection also enhanced the migration and invasion of human SW1353 cells. To extend these *in vitro* observations to the *in vivo* setting, mice were injected via tail vein with CH2879 chondrosarcoma cells treated with rhMMP-7 or transfected with either MMP-7 cDNA or the empty vector. rhMMP-7 treatment or ectopic expression of MMP-7 markedly increased lung colonization *in vivo*, as evidenced by the higher number of micrometastases and higher human DNA content in the lung of mice (Figs. 6A, 6B). To further support and confirm these *in vivo* data, live animal imaging experiments were carried out and CH2879 cells transfected with MMP-7 cDNA-mcherry plasmid were injected to mice via tail vein. The mice were anesthetized and scanned using Bruker *in vivo* imaging systems (MS FX PRO, Carestream, U.S.A). After 35 days injection, the cells migrated and colonized the lungs of NOD/SCID/IL2 receptor gamma knockout mice (Fig. 6C). Taken together, fluid shear stress induces the accumulation of cAMP- and IL-1β, which in turn activate PI3-K-, ERK1/2-, p38-dependent signaling pathways leading to MMP-7 induction via transactivation of NF-κB and c-Jun in human chondrosarcoma cells (Fig. 7); shear-induced MMP-7 promotes chondrosarcoma cell motility and invasion *in vitro* and *in vivo*.

## DISCUSSION

MMP-7 is one of the few MMPs that is secreted by tumor cells, but not stromal cells [22]. MMP-7 expression [7, 23, 24] correlates with the degree of malignancy in chondrosarcoma [7]. Interestingly, MMP-7 is localized at the invasive protrusions of tumor cells [7]. In line with these observations, we found that MMP-7 is overexpressed in human chondrosarcoma relative to appropriate normal controls. MMP-7 expression is also upregulated in shear-activated chondrosarcoma cells. In view of these findings and given that fluid shear is a relevant biomechanical signal in bone and cartilage (patho)physiology, we herein delineated the signaling pathways of MMP-7 induction in shear stress-activated human chondrosarcoma cells. Moreover, we report the critical roles of shear stress in promoting chondrosarcoma lung colonization in mice via the induction of the enzymatic activity of MMP-7 (Fig. 7).

Interstitial fluid flow in bone and cartilage is driven not only by mechanical loading but also by vascular pressure differences. Vascular pressure-driven interstitial fluid flow exists at all times in bone and cartilage because of transcortical pressure gradients [25], and is expected to be elevated in tumors in cancer patients [26]. Although interstitial flow moves around the cell-matrix interface in all directions rather than only on the apical side, both interstitial flow and shear fluid flow can drive cell responses via mechanical (i.e., shear stress on the cell surface) and non-mechanical effects (i.e., transport effects). It is noteworthy that shear fluid flow studies have been widely performed to investigate the mechanobiology of bone and cartilage-derived cells [25, 27].

MMPs have been implicated in the malignant transformation of human chondrocytes, and their expression is associated with chondrosarcoma recurrence [28, 29]. MMPs also promote the invasive potential of diverse tumor cell types [30–32]. Recent work reveals a link among fluid shear stress, MMP induction and cell invasion. For instance, Qazi *et al*. [33] showed that fluid shear stress enhances the invasive potential of glioma cells via regulating the activity of MMP-1 and MMP-2. We recently reported that shear-induced MMP-12 promotes the invasion and lung colonization of human chondrosarcoma cells [13]. High fluid shear stress has also the ability to modulate the mesenchymal transformation and invasion of aortic valve endothelial cells [34]. In line with these observations, Milkiewicz *et al*. [35] further established the link between shear stress and angiogenesis via stimulating the expression of VEGF in Sprague-Dawley rats. Besides tumor cells, fluid shear stress has ability to modulate the endothelial cell invasion into three-dimensional collagen matrices [36]. In agreement with these prior reports, our data reveal that shear stress exposure increases the lung colonization of human chondrosarcoma cells via MMP-7 activation (Fig. 1D).

MMP-7 is tightly regulated under physiological conditions and is elevated during the course of chondrosarcoma mesenchymal transformation [7]. Powell *et al.* [37] reported that MMP-7 overexpression increases the potential invasion of human prostate cancer cells (DU-145) into SCID mice. Witty *et al*. [38] also detected differences in the invasive potentials of MMP-7-transfected human colon cancer cells versus control cells. These *in vitro* findings were further corroborated by clinical observations showing that MMP-7 was overexpressed in six out of eight colon carcinomas [39]. Although there is no further evidence suggesting the direct relationship between MMP-7 and metastasis, a clear involvement of MMP-7 has been shown in tumor growth, invasion and spread via degrading its substrates including casein, elastin, laminin, proteoglycans, osteopontin, fibronectin and type IV collagen [40–42]. Nevertheless, MMP-7 might not regulate tumor cell metastasis via direct degradation of ECM. In some cases, human pro-MMP1, 2, and 9 can be activated by MMP-7, which further facilitate the metastasis of tumor cells [43–45]. Our data reveal that rhMMP-7 promotes the lung colonization of human chondrosarcoma cells (Fig. 7A). This finding was further substantiated by data showing that MMP-7 cDNA plasmid transfection enhanced the invasion and metastasis of human chondrosarcoma cells (Fig. 7B). Along these lines, MMP-7 activation may function as powerful machinery for ECM degradation, which facilitates local invasion and metastasis.

The underlying mechanisms of MMP-7 activation were not fully deciphered in previous investigations. We herein demonstrate the pivotal role of endogenous cAMP in fluid shear stress-induced MMP-7 synthesis, which was substantiated by the use of adenylate cyclase inhibitor SQ22536 that essentially abrogated MMP-7 induction in shear-activated chondrosarcoma cells (Fig. 2A). In line with our results, Hanemaaijer *et al*. [17] reported that forskolin affected the production of MMPs in a cell-type-specific way. However, they did not extend their study to MMP-7. Apart from cAMP, IL-1β has a critical role in inducing the expression of MMP-7 in shear-activated human chondrosarcoma cells (Fig. 2E). In line with our data, Klein *et al*. [15] demonstrated that IL-1β secreted from monocytic cells increases the expression of MMP-7 in the prostatic cell line LNCaP. Moreover, IL-1β is capable of inducing MMP-7 expression in human articular chondrocytes [16]. Therefore, both cAMP and IL-1β are key signaling molecules in mediating shear-induced MMP-7 expression in human chondrosarcoma cells.

We and others [14] have identified the two major MAPKs (ERK1/2, and p38) and PI3-K in transmitting cAMP- [46–48] and IL-1β-dependent signaling [47, 49], which result in MMP synthesis [50, 51]. In light of these findings [46–49, 52], we evaluated the potential contributions of these signaling pathways to shear-induced MMP-7 synthesis. Fluid shear stress activates PI3-K/AKT, ERK1/2, and p38 pathways in human chondrosarcoma cells via a cAMP- and IL-1β-dependent mechanism, as evidenced by the inhibition of AKT, ERK1/2 and p38 phosphorylation by a cAMP inhibitor and an anti-IL-1β neutralizing antibody. In addition, activation of PI3-K/AKT, ERK1/2, and p38 pathways leads to synthesis of MMP-7 in human SW1353 chondrosarcoma cells. In agreement with our data, several studies suggest that the ERK1/2 signaling pathway plays a key role in regulating MMP-7 expression. Kawabata *et al*. [53] demonstrated that transfection of ERK1/2 siRNA led to a significant reduction of pro-MMP-7 protein production in human HT-29 colorectal adenocarcinoma cells. In concert with this finding [53], ERK1/2 activation induces MMP-7 expression in human brain gliomas [54], colon [55] and pancreatic cancer cells [21, 56]. ERK1/2 activation could provide a source for upregulation of MMP-7 expression in human chondrosarcoma cells, but ERK1/2 activation may not be sufficient to achieve full activity of MMP-7. Apart from ERK1/2, PI3-K [57] and p38 [55, 58] have been involved in MMP-7 regulation in rat aortic vascular smooth muscle cells, human colon cancer and LOVO cells. Our data using pharmacological inhibitors and siRNA sequences specific for PI3-K, ERK1/2 and p38 reveal the pivotal role of these signaling pathways in mediating the effects of fluid shear stress on MMP-7 induction.

In light of our previous work showing that exposure of human chondrocytes to fluid shear stress transactivates the NF-κB p65 subunit [59, 60] and given that the expression of MMP-7 may be dependent on NF-κB [61], we evaluated the potential role of the NF-κB pathway in MMP-7 induction in human chondrosarcoma cells. We determined that fluid shear induces NF-κB phosphorylation at both Ser-276 and Ser-536. Moreover, inhibition of NF-κB markedly suppresses shear-induced MMP-7 expression, thereby establishing that the key role of canonical NF-κB pathway in this process. This finding is in concert with an earlier report suggesting a key role for NF-κB in the regulation of MMP-7 expression induced by helicobacter pylori infection in human gastric adenocarcinoma cells [62]. However, the NF-κB site may not be sufficient to achieve full transcription activity of MMP-7 synthesis. AP-1 (activator protein-1, formed by heterodimers of the two protooncogene families c-Jun and c-fos) has a binding site at the promoter region of MMP-7, which may cooperate with the NF-κB element to mediate fluid shear stress-dependent stimulation of MMP-7 transcriptional activity (Fig. 7). We herein report for the first time that both transcriptional factors are significantly stimulated by fluid shear stress, and in turn bind to their respective cis-elements, thereby inducing MMP-7 expression. This finding is further corroborated by our observations showing that PI3-K, ERK1/2 and p38 are able to regulate the phosphorylation of both NF-κB and c-Jun. In line with our data, c-Jun was also phosphorylated by PI3-K [63], ERK1/2 [64] and p38 [65] in human bronchial epithelial cells, mouse embryonic fibroblasts and human T lymphocytes. In addition, AP-1 has shown its pivotal roles in inducing MMP-7 expression in human gastric cancer cells [10]. Through the use of pharmacological inhibitors, promoter constructs, and ChIP assays, we demonstrated here the functional role of the NF-κB p65 subunit and c-Jun in shear-induced MMP-7 expression in human chondrosarocma cells.

In summary, we have elucidated the signaling pathway by which fluid shear stress upregulates the expression of MMP-7 in human chondrosarcoma cells. Specifically, fluid shear stress induces the synthesis of cAMP and IL-1β, which in turn activate PI3-K-, ERK1/2-, p38-dependent signaling pathways leading to MMP-7 induction via transactivation of NF-κB and c-Jun in human chondrosarcoma cells. Shear-induced MMP-7 potentiates chondrosarcoma cell motility and invasion, leading to the enhanced lung colonization of chondrosarcoma cells *in vivo*. These findings provide new insights into the mechanisms of MMP-7 upregulation in shear-activated human chondrosarcoma cells and might help us design better therapeutic strategies to combat chondrosarcomas.

## MATERIALS AND METHODS

### Reagents

Recombinant IL-1β, forskolin and the inhibitors LY294002, U0126, SB203580 and SP600125 were obtained from Sigma-Aldrich Corp. Antibodies specific for β-actin, AKT, p-AKT (Ser 473), ERK1/2, p-ERK1/2 (Thr 202/Tyr 204), p38, p-p38 (Thr 180/Tyr 182), c-Jun, p-c-Jun (Ser 63), NF-κB, p-NF-κB (Ser 536), p-NF-κB (Ser 276), IL-1β and siRNAs targeting AKT, ERK1/2, p38 were purchased from Cell Signaling Technology, Inc. (Danvers, MA, USA). Quinazoline (QNZ) was obtained from Enzo Life Sciences (Farmingdale, NY, USA). The IL-1β and cAMP enzyme immunoassay kits were from Cayman Chemical (Ann Arbor, MI). Recombinant human MMP-7 was obtained from R&D systems (Minneapolis, MN, USA). MMP-7 cDNA plasmids were purchased from Origene Technologies (Rockville, MD, USA). All reagents for qRT-PCR and SDS-PAGE experiments were purchased from Bio-Rad Laboratories. All other reagents were from Invitrogen (Carlsbad, CA) unless otherwise specified.

### Cell culture and shear stress exposure

SW1353, HS.819.T and CH2879 cells were grown (37°C in 5% CO_2_) on glass slides in DMEM/F12 medium supplemented with 10% FBS as previously described [59, 66–69]. Before shear exposure, cells were incubated for 18 h in serum-free medium supplemented with 1% Nutridoma-SP (Roche), a low serum replacement [70, 71]. Cells were then subjected to a shear stress level of 2 dyn/cm^2^ for prescribed periods of time in medium containing 1% Nutridoma-SP, using a streamer gold flow device (Flexcell International, Hillsborough, NC). In select experiments, the pharmacological agents were added to the medium at the indicated concentrations just before the onset of shear exposure. In separate experiments, SW1353 cells were seeded on 6-cm tissue culture dishes (10^6^ cells per dish) in DMEM/F12 medium supplemented with 10% FBS [69]. 24 h later, human chondrosarcoma cells were cultured in serum-free medium for another 24 h before being incubated with select inhibitors in the presence or absence of forskolin or IL-1β.

### Quantitative Real-Time PCR (qRT-PCR)

qRT-PCR assays were performed on the iCycler iQ detection system (Bio-Rad) using total RNA, the iScript one-step RT-PCR kit with SYBR green (Bio-Rad) and primers. The GenBank accession numbers and forward (F-) and reverse (R-) primers are as follows:

MMP-7 (NM_002423), F-ATGTGGAGTGCCA GATGTTG, R-GCCAATCATGATGTCAGCAG;

The GenBank accession numbers and forward (F-) and reverse (R-) primers for GAPDH are provided in our previous publications [59, 69]. GAPDH was used as internal control. Reaction mixtures were incubated at 50°C for 15 min followed by 95°C for 5min, and then 35 PCR cycles were performed with the following temperature profile: 95°C 15s, 58°C 1 min, 68°C 1 min, 77°C 20s. Data were collected at the (77°C 20 s) step to remove possible fluorescent contribution from dimer-primers [59, 69]. Gene expression values were normalized to GAPDH.

### Western blot analysis

SW1353 or HS.819.T cells from static and sheared specimens were lysed in a radioimmune precipitation assay buffer (25 mM Tris•HCl pH 7.6, 150 mM NaCl, 1% NP-40, 1% sodium deoxycholate, 0.1% SDS) containing a cocktail of proteinase inhibitors (Pierce Chemical Company). The protein content of the cell lysates was determined using bicinchoninic acid (BCA) protein assay reagent (Pierce Chemical Company). Total cell lysates (4 μg) were subjected to SDS-PAGE, transferred to a membrane, and probed with a panel of specific antibodies. Each membrane was probed using one antibody only. β-actin was used as loading control. All Western hybridizations were performed at least in triplicate using a different cell preparation each time.

### Measurement of cAMP and IL-1β concentration in medium

cAMP and IL-1β levels in both static and sheared medium were determined using the corresponding kits following the manufacturer's instructions [14]. The concentration of total protein in the medium was used as loading control, and the results were expressed as pmol cAMP or pg IL-1β per μg of total protein.

### Transfection

SW1353 chondrosarcoma cells were transfected with 100 nM of a siRNA oligonucleotide sequence specific for AKT, ERK1/2 or p38. In control experiments, cells were transfected with 100 nM of scramble siRNA. In promoter assays, SW1353 chondrosarcoma cells were transfected with 1.6 μg/slide of the MMP-7 promoter reporter construct together with the pRL-SV40 vector. Transfected cells were allowed to recover for at least 12 h in growth medium, and then incubated overnight in medium containing 1% Nutridoma-SP before their exposure to shear stress. In live animal imaging experiments, CH2879 cells were transfected with MMP-7 cDNA-mcherry plasmids and further established stable transfected cell lines for tail vein injection.

### Luciferase promoter constructs

A 2, 036-base pair (bp) MMP-7 promoter construct, corresponding to the sequence from −1997 to +39 relative to the transcription start site of the 5′-flanking region of human MMP-7 gene, was generated from human genomic DNA, using specifically designed forward 5′- TATCGATAGGTACC**GAGCTC**GGTAAGATGACTGTTAAGG-3′ and reverse 5′- GATCGCAGAT**CTCGAG**CCAGAGACAATTGT TCTTGG-3′ primers incorporating *Sac 1* and *Xho1* restriction sites (underlined) at the 5′ ends and 3′ ends, respectively [20]. The amplicon was then inserted upstream of the luciferase reporter gene in the pGL3-basic vector (Promega). The various truncations were generated using designed primers, incorporating the relevant restriction enzyme sites (underlined) to allow for proper orientation upstream of the luciferase reporter: −1597 bp construct forward 5′- TATCGATAGGTACC**GAGCTC**AGGATTACA GGCGTGAGCC-3′, the –1199 bp construct forward 5′- TATCGATAGGTACC**GAGCTC**GCTCCAGCAT ATTTGGAGTG-3′, and the –579 bp construct forward 5′- TATCGATAGGTACC**GAGCTC**TATAGAGTGGCC ACTAATCC-3′. All constructs were sequenced and confirmed to be identical to those reported for the MMP-7 promoter. Point mutations within various transcription factor-binding sites were performed, using standard site-directed mutagenesis methodologies within the 2100 bp construct. For the NF-κB, a series of site mutation constructs was made as following: mNF-κB, 5′-GAAATGCCT-3′ to 5′-GA**T**ATGCC**C**-3′. In addition, AP-1 site mutation constructs were made as following: mAP-1, 5′-ATGAGTCACCT-3′ to 5′-ATG**C**GT**T**ACCT-3′. All construct sequences were confirmed using DNA sequencing.

### Promoter assay

Firefly and *Renilla* luciferase activities were measured by use of the Dual-Luciferase Report Assay kit (Promega). Firefly luciferase activities were normalized to the *Renilla* luciferase controls. Data are expressed as ratios of shear to static normalized firefly luciferase activity unless otherwise stated.

### EMSA and supershift assay

A 5′-biotinylated oligonucleotide probes (5′-GGAAATGCCTT-3′; 5′-ATGAGTCACCT-3′) were synthesized containing the NF-κB and c-Jun cis-element present on the MMP-7 promoter. EMSAs were performed with a commercially available nonradioisotopic EMSA kit (LightShift Chemiluminescence EMSA kit; Pierce). Briefly, nuclear extracts (1–2 μg) were incubated in 10 × binding buffer (supplemented with 50 ng of poly (dI-dC), 2.5% glycerol, 0.05% Nonidet P-40, 5 mM MgCl_2_, and 0.25 mg of bovine serum albumin), containing 20 fmol of biotinylated, double-stranded probes for NF-κB or c-Jun for 30 min on ice. For competition binding, a 200-fold excess of unlabeled (cold) probe was incubated with nuclear extracts before the inclusion of the biotinylated one. For supershift assays, the nuclear extracts were preincubated for 30 min on ice with an anti-p65 antibody. The biotinylated oligonucleotide probe specific for NF-κB or c-Jun was then added to the reaction mixture and incubated for another 30 min on ice. To exclude the possibility of nonspecific binding, a 5′-biotinylated random probe (5′- AGTCCGTTGAA-3′; 5′-GAGCATTATCC-3′) designed using a random sequence generator was used in shift and supershift assays. The protein-DNA complexes were resolved on a native 6% polyacrylamide retardation gel in 0.5 × Tris borate-EDTA running buffer at 10 mA for 1 h, transferred to a nylon membrane (Pierce), visualized using the LightShift Chemiluminescence kit (Pierce) and exposed to Kodak x-ray film (Pierce).

### Chromatin immunoprecipitation (CHIP)

This assay was performed using the EZ ChIP kit following the manufacture's instructions (Upstate Biotechnology) as previously described [69, 72, 73]. Forward (F-) and reverse (R-) primers for MMP-7 promoter amplification by qPCR are as follows: NF-κB, F-AGGATTACAGGCGTGAGCC, R-GACATGTGATAAGGTGCACC; AP-1, F-GTGTGCTTCCTGCCAATAAC, R-CCAGAGACAA TTGTTCTTGG.

### Casein zymography

Activity of MMP-7 was determined according to the method previously described [18]. In brief, the conditioned media of cells were collected and mixed with non-reducing sample buffer containing 0.5 M Tris (pH 6.8), 5% SDS, 20% glycerol, and 1% bromphenol blue in a 1:1 ratio and electrophoresed directly on 10% SDS-polyacrylamide gels (SDS-PAGE). After electrophoresis, gels were washed for 1 h at room temperature in a 2.5% (v/v) Triton X-100 solution to remove SDS, transferred to zymogram development solution (10 mM CaCl_2_, 50 mM Tris–HCl, pH 7.4, and 0.02% NaN_3_), and incubated for 72 h at 37°C. Gels were stained for 30 min with 0.1% (w/v) Coomassie brilliant blue (CBB) in 50% (v/v) methanol/10% (v/v) acetic acid and destained in 20% (v/v) methanol /10% (v/v) acetic acid. Areas of lysis were observed as white bands against a black background.

### Transwell migration and invasion assay

5 × 10^4^ SW1353 cells treated with 10 μM forskolin, 100 ng/ml IL-1β or vehicle controls were seeded on 24 well transwell inserts with 8 μm pore (Becton Dickinson, Bedford, MA, USA) in the absence or presence of LY294002 (10 μM), U0126 (10 μM), SB203580 (10 μM), SP600125 (10 μM) or QNZ (2 μM). In select experiments, SW1353 cells were treated rhMMP-7 (1 μg/ml) or vehicle controls. The lower chamber contains medium supplemented with 10% FBS, which serves as chemoattractant. 48 h later, cells on the upper side were removed with a cotton swab. Then, the membrane was fixed with 3.7% formaldehyde and stained with 0.1% crystal violet in PBS (−). Under low magnification microscope (× 100), five horizons (up, down, left, right, center) were examined on each membrane. The number of cells went through the polycarbonate membrane was counted and averaged for each field of vision to indicate the migratory ability of tumor cells. In invasion experiments, the polycarbonate membrane was pre-coated with Matrigel (Becton Dickinson, Bedford, MA, USA) before seeding SW1353 cells in the upper inserts. Following the same protocol as described above, the number of cells, which went through the matrigel, is indicative of their invasive capacity.

### Immunohistochemistry

Human bone tissue microarrays (US Biomax Inc., Rockville, MD, USA) included 69 chondrosarcoma tissue cores and 24 normal bone tissue cores were fixed on tissue microarray slides. Slides were first deparaffinized with xylene, rehydrated in a graded series of ethanol and submerged in 3% hydrogen peroxide to eliminate endogenous peroxidase activity. MMP-7 level was determined using immunohistochemical staining kit, following the manufacturer's instructions (Invitrogen, Carlsbad, CA, USA).

### Tail vein injections

NOD/SCID/IL2 receptor gamma knockout mice (10 per experimental group; obtained from Jackson Lab, Strain 005557) were injected via the tail vein with 10^5^ CH2879 chondrosarcoma cells in a volume of 100 μl of serum free medium followed by tail vein injection with either recombinant human MMP-7 (100 μl of 10 μg/ml) or vehicle control at the day of the cell injection (*t* = 0 weeks) and weekly thereafter for 5 weeks. In select experiments, cells were transfected with MMP-7 cDNA or MMP-7 cDNA-mcherry plasmids before injecting to the tail vein of mice. In other experiments, CH2879 cells, exposed to either static or shear (2 dyn/cm^2^) conditions for 48 h, were suspended in their corresponding conditioned medium and injected into mice via tail vein (*t* = 0 weeks); the conditioned medium was injected via the tail vein every 3 days for 5 weeks. Mice were euthanized 5 weeks post cell injection.

### Luciferase assays and live animal imaging

The CH2879 cells transfected with MMP-7 cDNA-mcherry plasmids were injected to mice via tail vein. After 35 days post cell injection, mice were anesthetized and scanned using Bruker *in vivo* imaging systems (MS FX PRO, Carestream, U.S.A).

### Histopathology of lung tissue

Lung samples for pathology were fixed in 10% buffered formalin. The left lung lobe was used for *hLINE* analysis and the remaining lobes were embedded in a single cassette and sectioned for histopathological analysis of metastatic foci [74, 75]. Samples were sectioned by cryostats at 10 μm, and stained with hematoxylin and eosin using standard techniques. Tissue collection, histopathology analysis, and grading were performed by a pathologist (DLH). Metastatic foci were counted in 3 lung histology sections from each mouse and scored as follows: no lung colonization = 0 foci; mild = 1–10 foci; moderate = 11–20 foci; severe > 20 foci.

### Quantification of human Long Interspersed Nuclear Element-1 (hLINE-1) gene

DNA was extracted from mouse lung tissue as previously described [74, 75] using the DNeasy blood and tissue kit (Qiagen, Valencial, CA, USA) in a sterile biological safety cabinet to minimize the risk of human DNA contamination. Elutions were analyzed via qPCR as reported previously [74, 75]. Briefly, qPCR was performed in 15 μl volume with the following components: 7.5 μl iQ SYBR Green Supermix (Bio-rad, Hercules, CA, USA), 1.5 μl of each 10 μM forward (5′-TCACTCAAAGCCGCTCAACTAC-3′) and reverse (5′-TCTGCCTTCATTTCGTTATGTACC-3′) primers, and 4.5 μl of purified DNA. The reaction was monitored on an iCycler/iQ5 (Bio-Rad) with the following cycles: (94°C, 2 min) × 1, (94°C, 10 s; 67°C, 15 s; 70°C, 15 s) × 3, (94°C, 10 s; 64°C, 15 s; 70°C, 15 s) × 3, (94°C, 10 s; 61°C, 15 s; 70°C, 15 s) × 3, and (94°C, 10 s, 59°C, 15 s; 70°C, 15 s) × 35. Threshold cycle number was calculated using Bio-Rad iQ5 software. Dilutions of human DNA purified from chondrosarcoma cells were included in each plate to serve as standards.

### Statistics

Data represent the mean ± S.E. of at least 3 independent experiments. Statistical significance of differences between means was determined by Student's *t*-test or one-way ANOVA, wherever appropriate. If means were shown to be significantly different, multiple comparisons by pairs were performed by the Tukey test [71].

## SUPPLEMENTARY FIGURES


