# Supplementary material for: By activating matrix metalloproteinase-7, shear stress promotes chondrosarcoma cell motility, invasion and lung colonization
Source: Oncotarget. 2015 Mar 14;6(11):9140–59. doi: 10.18632/oncotarget.3274 (PMC4496208; doi:10.18632/oncotarget.3274)
Supplement: Supplementary file 1 [file oncotarget-06-9140-s001.pdf]

SUPPLEMENTARY FIGURES

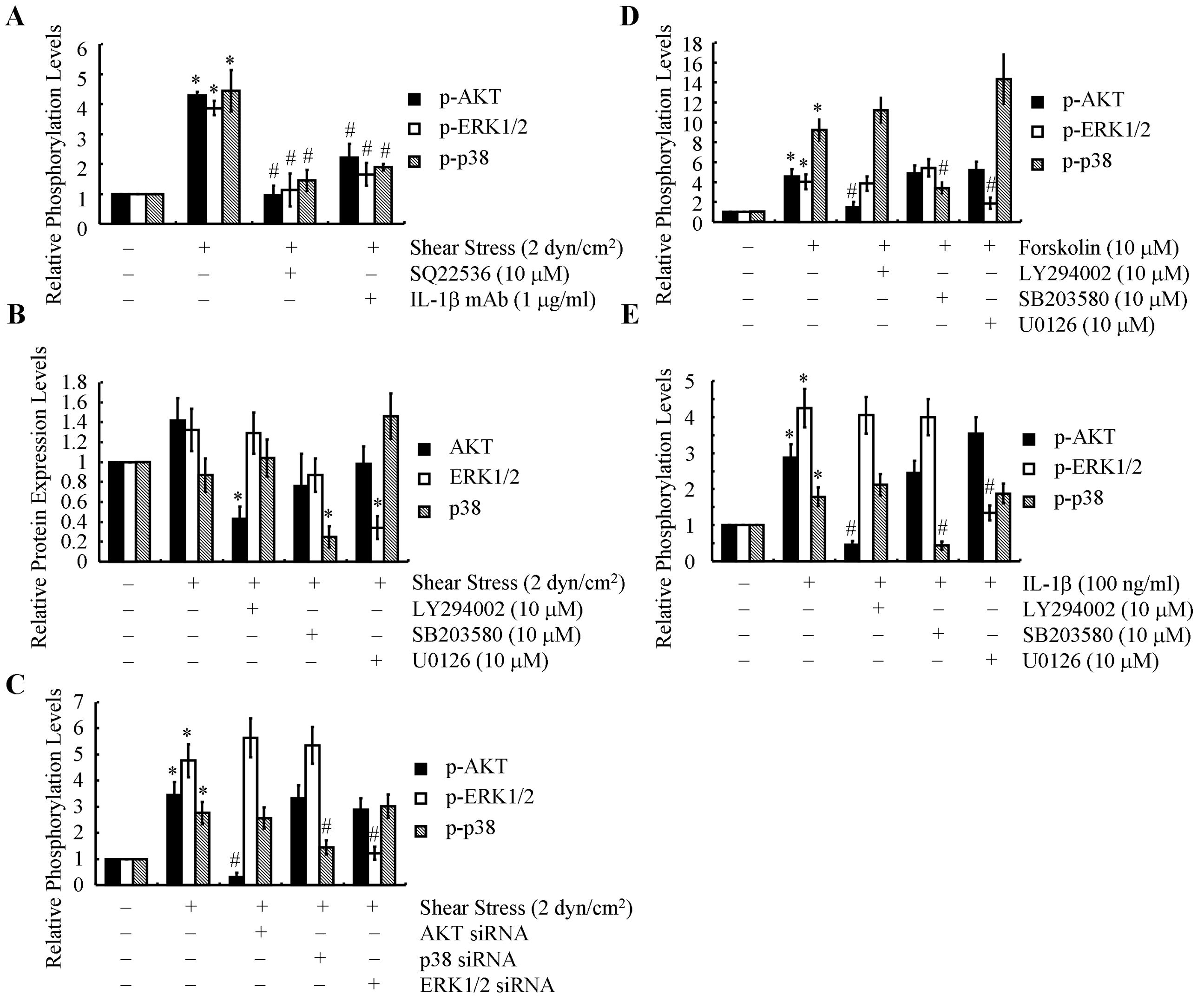

Supplementary Figure S1: The Statistical analysis of western blots in Figure 2.

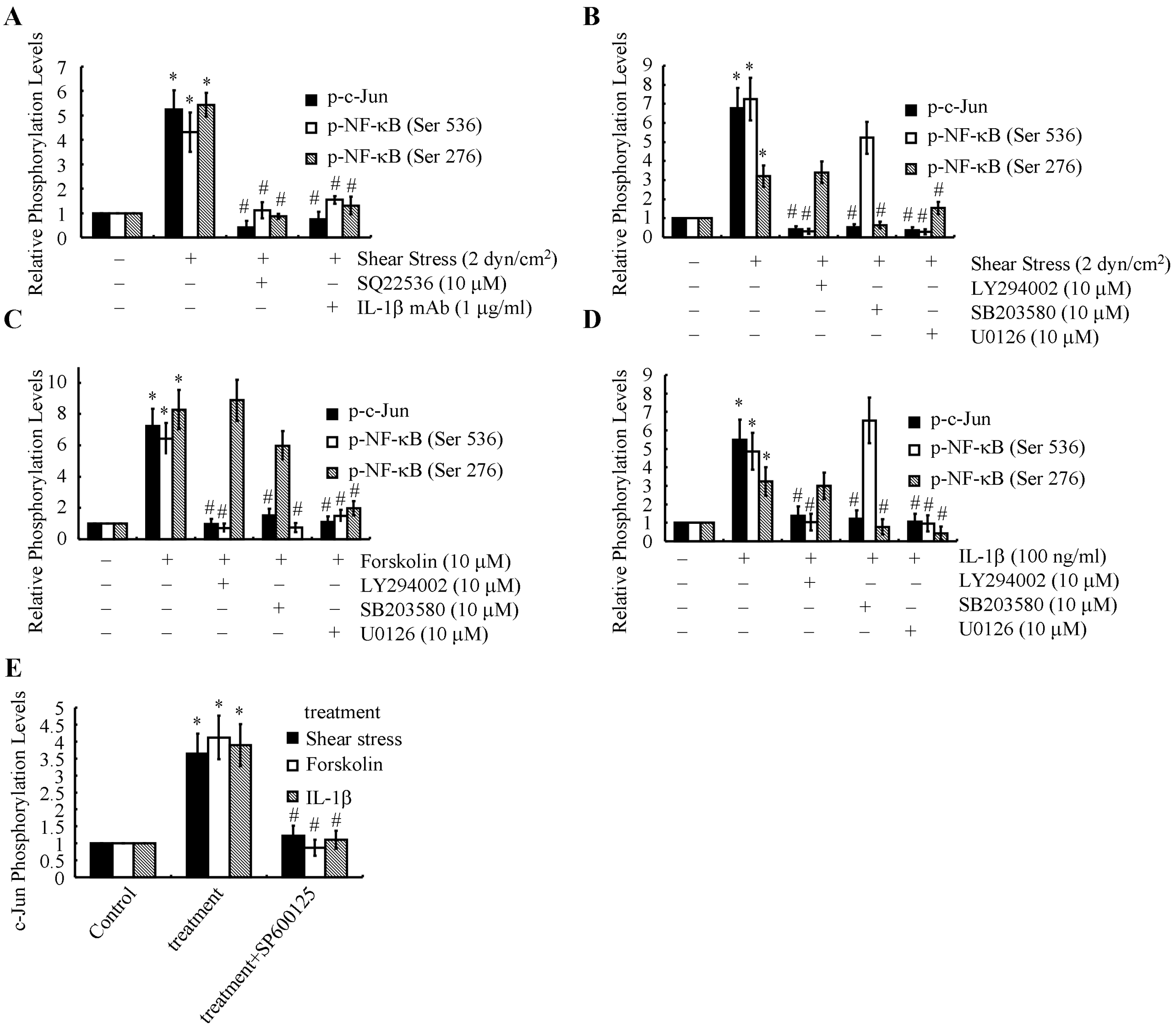

Supplementary Figure S2: The Statistical analysis of western blots in Figure 3.
